# MADD Knock-Down Enhances Doxorubicin and TRAIL Induced Apoptosis in Breast Cancer Cells

**DOI:** 10.1371/journal.pone.0056817

**Published:** 2013-02-15

**Authors:** Andrea Turner, Liang-Cheng Li, Tania Pilli, Lixia Qian, Elizabeth Louise Wiley, Suman Setty, Konstantin Christov, Lakshmy Ganesh, Ajay V. Maker, Peifeng Li, Prasad Kanteti, Tapas K. Das Gupta, Bellur S. Prabhakar

**Affiliations:** 1 Department of Microbiology and Immunology, College of Medicine, University of Illinois at Chicago, Chicago, Illinois, United States of America; 2 Department of Pathology, College of Medicine, University of Illinois at Chicago, Chicago, Illinois, United States of America; 3 Department of Surgical Oncology, College of Medicine, University of Illinois at Chicago, Chicago, Illinois, United States of America; Aix-Marseille University, France

## Abstract

The Map kinase Activating Death Domain containing protein (MADD) isoform of the *IG20* gene is over-expressed in different types of cancer tissues and cell lines and it functions as a negative regulator of apoptosis. Therefore, we speculated that MADD might be over-expressed in human breast cancer tissues and that MADD knock-down might synergize with chemotherapeutic or TRAIL-induced apoptosis of breast cancer cells. Analyses of breast tissue microarrays revealed over-expression of MADD in ductal and invasive carcinomas relative to benign tissues. MADD knockdown resulted in enhanced spontaneous apoptosis in human breast cancer cell lines. Moreover, MADD knockdown followed by treatment with TRAIL or doxorubicin resulted in increased cell death compared to either treatment alone. Enhanced cell death was found to be secondary to increased caspase-8 activation. These data indicate that strategies to decrease MADD expression or function in breast cancer may be utilized to increase tumor cell sensitivity to TRAIL and doxorubicin induced apoptosis.

## Introduction

Map kinase Activating Death Domain containing protein (MADD), a splice variant of the *IG20* gene, is essential for cancer cell survival and confers resistance to tumor necrosis factor-related apoptosis-inducing ligand (TRAIL) treatment. TRAIL normally binds to death receptors-4 (DR4) and -5 (DR5) on cancer cells resulting in DR oligomerization and subsequent recruitment of the Fas associated Death Domain containing protein (FADD) and procaspase-8 to DRs [1]–[3]. Procaspase-8 undergoes proximity induced activation and cleavage forming caspase-8 which then activates the executioner caspase-3 that causes cell death. However, in cancer cells where MADD is over-expressed, MADD binds to DR4 and DR5 and prevents FADD recruitment to the DRs. Upon MADD knockdown, FADD is more readily recruited to the DRs and results in enhanced apoptosis [4], [5].

TRAIL is unique in that it generally does not adversely affect normal cells or tissues [6]. Recent studies have shown that low concentrations of doxorubicin can sensitize cancer cells to TRAIL-induced apoptosis. The ability of doxorubicin to synergize TRAIL-induced apoptosis demonstrates a critical interplay between the extrinsic and the intrinsic apoptotic pathways [7]–[10] that can be exploited to more effectively kill cancer cells while reducing the undesirable side effects of high dose chemotherapy. However, development of chemotherapy and TRAIL resistance due to the expression of different anti-apoptotic proteins remains a major challenge.

Our earlier studies have shown that MADD is one such anti-apoptotic protein[5]. MADD is expressed at much higher levels in cancer cells and tissues relative to their normal counterparts. It binds to DR4 and DR5 and confers resistance to TRAIL induced apoptosis in thyroid, ovarian and cervical cancer cell lines [4], [11]–[13]. However, neither the levels of expression of MADD in breast cancer tissues nor its ability to confer resistance to chemotherapeutic or TRAIL induced apoptosis in breast cancer cells has been investigated. Therefore, we examined MADD expression in breast cancer tissues and tested the effects of MADD knockdown on TRAIL and doxorubicin induced apoptosis of breast cancer cells.

## Results

### Endogenous MADD is highly expressed in breast cancer tissues

To determine if MADD is expressed differentially we stained breast cancer tissue microarrays using a MADD reactive antibody [14]. MADD protein expression could be evaluated in 56% (25/44) of normal tissues, in 87% (34/39) of DCIS cases and in 95% (82/86) of invasive carcinomas. Absence of target lesion in tissue cores or loss of tissue during the sectioning or staining contributed to the reduction in the number of tissues that were evaluated for MADD expression. The majority of normal breast tissues were negative or weakly positive while the DCIS (p = 0.01) and the IBC (p = 0.001) cases, were moderately or strongly positive (Fig. 1).

**Figure:**
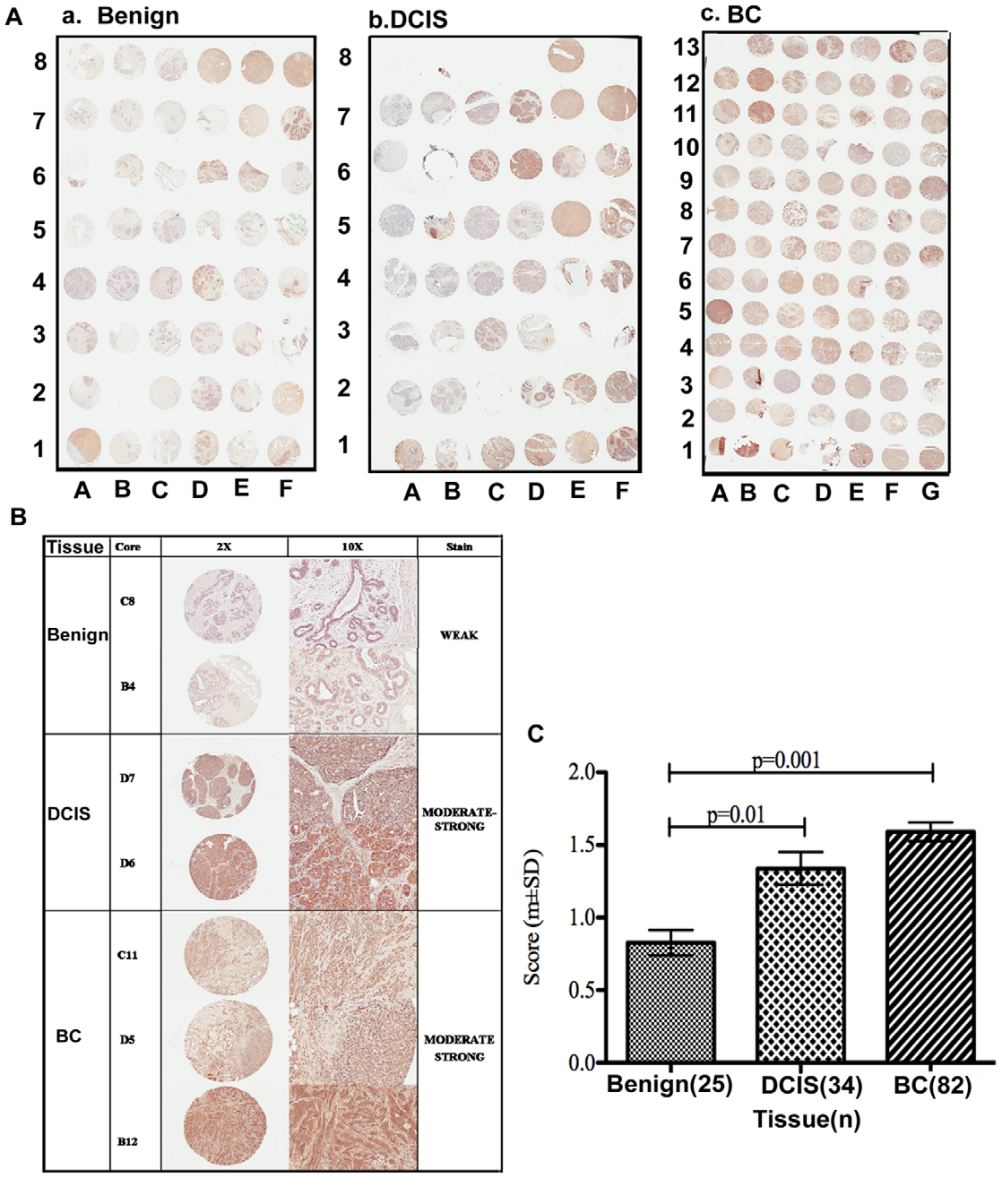
Expression of MADD protein in breast cancer tissues. **A.** Tissue microarrays (TMA) containing tissue sections representing benign breast lesions, ductal carcinoma *in situ* (DCIS) and invasive breast carcinomas (IBC) were prepared and stained for MADD expression. **B.** The TMAs were scored for the degree of MADD expression by two independent investigators in a semi-quantitative fashion (0 = negative, 1 = weak intensity, 2 = moderate intensity, 3 = strong intensity). **C.** Statistical analysis was carried out using one-way ANOVA with Tukey-Kramer post-hoc as described under materials and methods. A significant difference in the intensity of MADD stain in DCIS and IBC cases as compared to normal tissues was noted (p = 0.01 and p = 0.001 respectively).

### MADD is highly expressed in breast cancer cells and can be selectively knocked down by small hairpin-RNAs (sh-RNAs)

**Figure 2:**
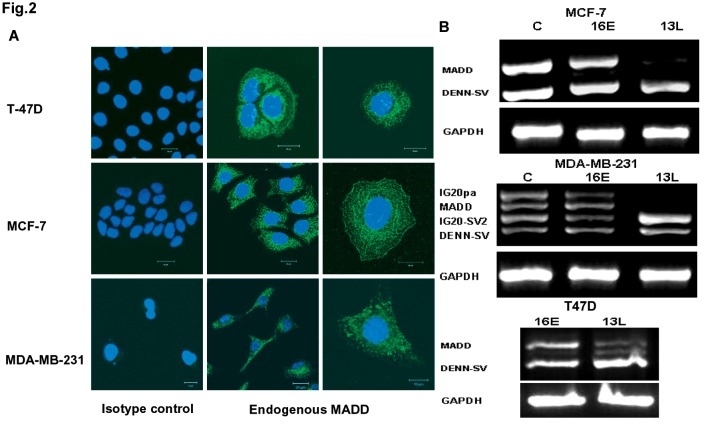
Expression of MADD, and selective knock down of IG20 isoforms in breast cancer cells. **A.** shows immunofluorescence staining of MADD in MCF-7, MDA-MB-231 and T47D breast cancer cell lines. **B.** Selective knockdown of *IG20* isoforms in breast cancer cell lines. Our previously generated 16E and 13L-shRNA lentivirus [5] can efficiently knock down the appropriate isoforms in MCF-7, MDA-MB-231 and T47D cells. All three cell lines were transduced with the indicated virus for 72 h at which point RNA was extracted and used (1μg) for RT-PCR using F2-B2 primers. The products were run on a 2% agarose gel to separate and visualize the isoforms.

MADD expression was determined by immunofluorescence staining using an exon 13L-specific antibody in three breast cancer cell lines (i.e. MCF-7, MDA-MB-231 and T47D cells) (Fig. 2A). Our previously generated shRNAs were used at a transduction efficiency of over 70% as determined by Green Fluorescent Protein (GFP) expression (not shown). The 13L-shRNA targeted exon 13L and selectively down-modulated IG20pa and MADD in MDA-MB-231 cells, which expressed all four *IG20* isoforms, and MADD alone in MCF-7 and T47D cells, which expressed only MADD and Differentially Expressed in Normal and Neoplastic tissues Splicing Variant (DENN-SV) isoforms (Fig. 2B). In contrast, the 16E-shRNA that specifically targets exon 16 down-modulated IG20pa by over 62% and IG20-SV2 over 55% in MDA-MB-231 cells, and had no apparent effect on the other two cell lines (Fig. 2B). Unlike the SCR-shRNA, which had little or no effect on the expression of *IG20*-SVs relative to untreated controls, 16E shRNA specifically targeted IG20pa and IG20-SV2 and allowed for MADD expression. Therefore, we used 16E-shRNA as a more appropriate negative control in all our subsequent experiments.

### Down-modulation of MADD in breast cancer cells leads to spontaneous apoptosis

**Figure 3:**
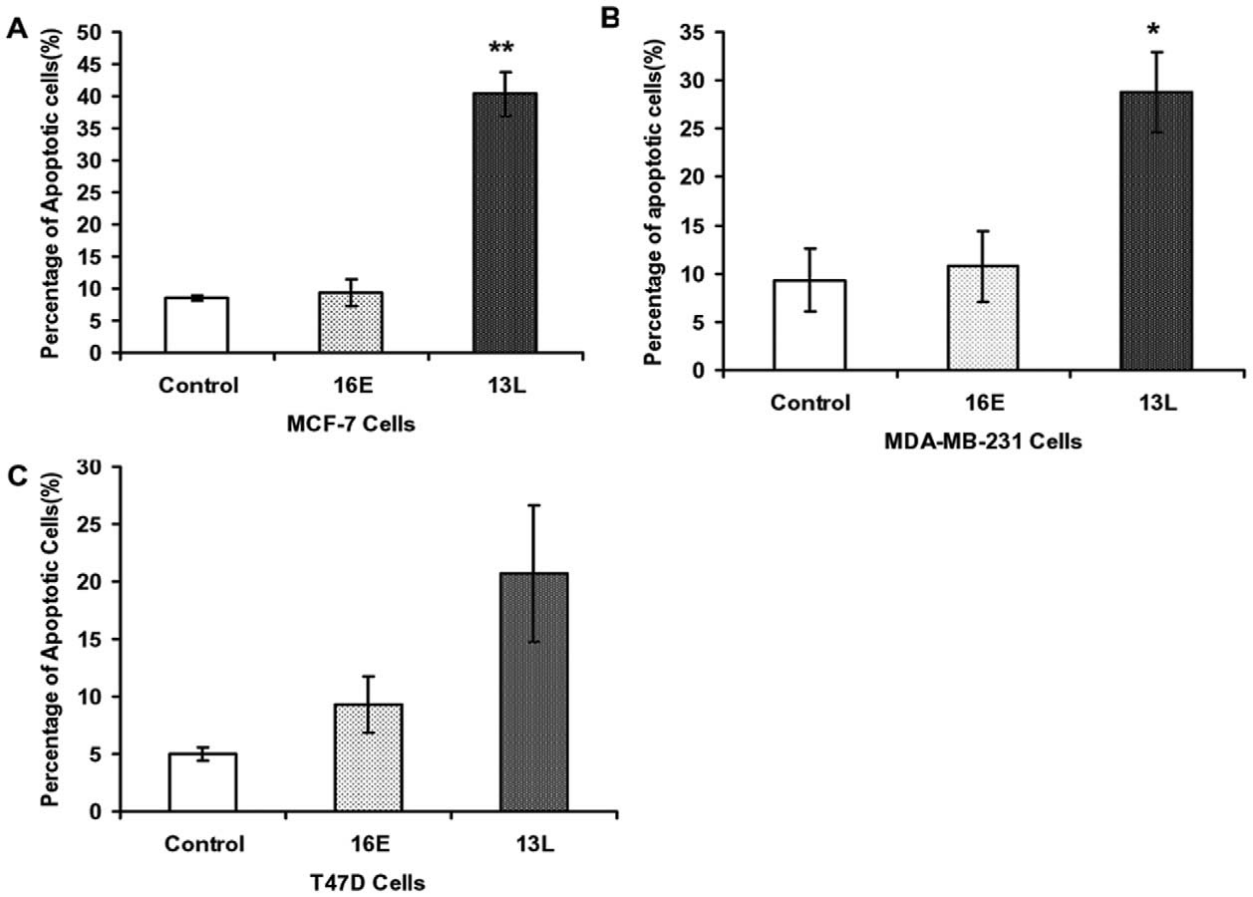
MADD knockdown in breast cancer cells results in spontaneous apoptosis. MCF-7 (**A**), MDA-MB-231(**B**) and T47D(**C**) breast cancer cells were transduced for 72 h with indicated viruses, they were then stained with TMRM, harvested and subjected to flow cytometry to determine the percentage of apoptotic cells. *p<0.05;** p<0.01 13L shRNA *vs.* 16E shRNA. Summarized data from three independent experiments are shown.

Targeting exon 13L with 13L-shRNA resulted in apoptosis in MCF-7 (P<0.001; compare 13L *vs* 16E induced apoptosis) and MDA-MB-231 (P<0.05; compare 13L *vs* 16E induced apoptosis) cells (Fig. 3 A–B), but not in T47D cells (P>0.05; compare 13L *vs* 16E induced apoptosis) (Fig. 3C). Down modulation of IG20pa and IG20-SV2 isoforms using 16E-shRNA had no effect on cell apoptosis.

Relative to control cells and cells treated with 16E-shRNA, cells expressing 13L-shRNA showed a significant reduction in their numbers (Fig. S1 A, B). To differentiate whether the decreased number was due to cell death or their inability to proliferate, we plated equal number of cells expressing various shRNAs, and the number and size of colonies were determined. Although significantly fewer colonies were formed when cells were transduced with 13L-hRNA, the size of the colonies was not different from those formed in both MCF-7 and MDA-MB-231 control cells (Fig. S1 C, D). Together, these results indicated that the primary effect of MADD knockdown was to enhance spontaneous apoptosis.

### Effect of TRAIL and doxorubicin on apoptosis in breast cancer cells with and without MADD knockdown

**Figure 4:**
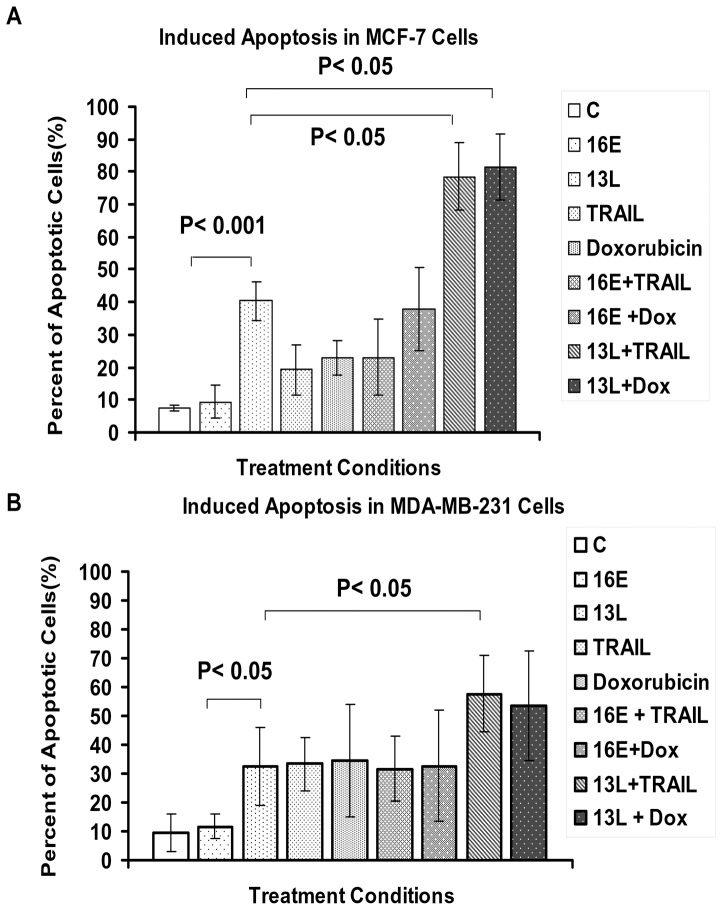
Upon MADD knockdown, low doses of TRAIL and doxorubicin can enhance apoptosis. MCF-7 (**A**) and MDA-MB-231 (**B**) cells were treated with TRAIL for 24 h and with doxorubicin for 72 h at a concentration of 10 ng/ml. Cells were stained with TMRM and analyzed by FACS. Statistical significance levels are shown on the figures. Summarized data from three independent experiments are shown.

Next, we examined the apoptotic effects of TRAIL and doxorubicin on MCF7 and MDA-MB-231 cells in the presence or absence of MADD expression. MCF-7 and MDA-MB-231 cells were transduced with indicated lentiviruses for 72 hours and treated concurrently for 24 hours or 72 hours with suboptimal doses of TRAIL or doxorubicin that were at least 10 times lower than those used in previous studies [15]–[19]. Both MCF-7 and MDA-MB-231 cells showed significant increases in spontaneous apoptosis upon MADD knockdown relative to control cells (Fig. 4A, B, compare 13L *vs.* 16E induced apoptosis; P<0.001 in MCF-7, and P<0.05 in MDA-MB-231). Furthermore, combining MADD knockdown with TRAIL treatment resulted in enhanced apoptosis in both cell types (Fig. 4A and 4B; P<0.05, 13L-shRNA transduced cells treated with TRAIL *vs.* untreated cells). MADD knockdown combined with doxorubicin treatment significantly increased apoptosis in MCF-7 cells (P<0.05, Fig. 4A), but not in MDA-MB-231 (Fig. 4A). These results suggested that MADD knockdown can enhance cell death induced by either TRAIL or doxorubicin treatment.

### Doxorubicin treatment promotes death receptor expression

To evaluate if doxorubicin treatment affected DR expression, we treated cells with doxorubicin (10 ng/ml) for up to 72 hours and determined DR expression on their surface. We did not find any increase in the expression of decoy receptors in both cell lines (data not shown). As shown in Fig. S2, the levels of DR4 and DR5 on the surface of MCF-7 cells were increased starting at 48 hrs after doxorubicin treatment which reached significantly higher levels after 72 hrs (Fig. S2A, C). However, only the expression of DR5 was increased to a significant level on the surface of MDA-MB-231 cells 72hrs after the treatment as compared to untreated cells (Fig. S2D).

### MADD knockdown results in enhanced extrinsic apoptosis

**Figure 5:**
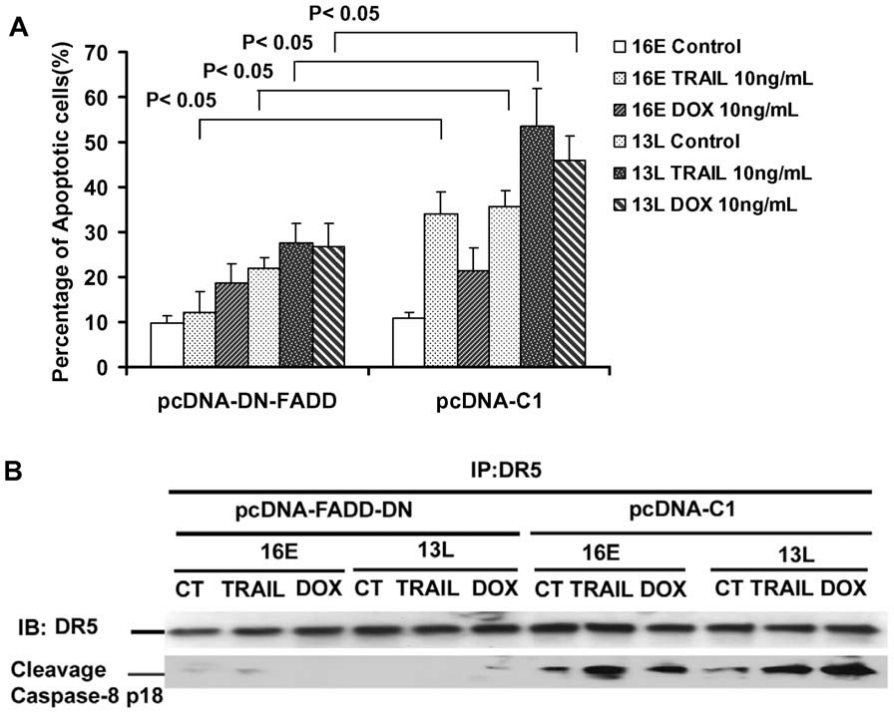
MADD Knockdown, followed by treatment with TRAIL or doxorubicin, results in increased caspase-8 activation. MADD knockdown in combination with either TRAIL or doxorubicin results in extrinsic apoptosis. MCF-7 cells were transfected with either empty vector (pcDNA) or pcDNA-DN-FADD plasmid, Twenty four hours later the cells were transduced with 16E or 13L lentivirus for 72 hours and were left alone, or treated with TRAIL or doxorubicin. One third of the cells were stained with TMRM and subjected to FACS analysis to determine apoptosis (**A**), and the other two thirds were used for DR5 immunoprecipitation (**B**). Separated immune complexes were immunoblotted using antibodies specific for cleaved caspase-8 and DR5. Summarized data from three independent experiments are shown.

To determine if increased activation of the extrinsic apoptotic pathway contributed to increased death in cells devoid of MADD expression, we blocked the extrinsic pathway (i.e. prevent capase-8 activation) by expressing a dominant negative form of FADD (DN-FADD) in MCF-7 cells. As seen in Fig. 5A, the DN-FADD abrogated cell death indicating that indeed activation of the extrinsic pathway was essential for the increased apoptosis noted in cells that were devoid of MADD expression and treated with either TRAIL or doxorubicin.

MADD knockdown resulted in DR5 associated caspase-8 activation (i.e. increased cleaved caspase-8; p18), which was further enhanced when MADD knockdown was coupled with TRAIL or doxorubicin treatment (Fig. 5B), while caspase-8 cleavage was abrogated in the presence of DN-FADD (Fig. 5B). Collectively, these results indicated that activation of the extrinsic pathway was critical not only for enhanced TRAIL-induced apoptosis but also for doxorubicin-induced apoptosis in breast cancer cells with MADD knock down.

## Discussion

We have previously shown enhanced levels of expression of MADD in multiple cancer tissues [5], [11], [13], [20] including a limited number of breast cancer tissues [21]. Here, we have demonstrated that the pro-survival protein MADD, an isoform of the *IG20* gene, is significantly over expressed in breast tissues containing DCIS and invasive breast cancer, as well as in the three evaluated breast cancer cell lines. We confirmed the ability to down regulate MADD expression using shRNA, which resulted in spontaneous apoptosis that was further augmented by treatment with TRAIL or doxorubicin, a standard chemotherapeutic agent used in advanced invasive breast cancer patients. In the setting of MADD knockdown, TRAIL induced apoptosis was as effective as that induced by doxorubicin.

Previous studies from our laboratory that employed selective knockdown of *IG20* splice variants using exon-specific shRNAs have shown that the MADD isoform of the *IG20* gene is required and sufficient for cancer cell survival [5]. Further, we have shown that treatment of cervical (HeLa), ovarian, (PA-1) and thyroid (WRO) cancer cells devoid of expression of the endogenous MADD are more susceptible to both spontaneous and TRAIL- and TNFα-induced apoptosis [5], [11]–[13], primarily through the activation of the extrinsic apoptotic pathway. The extrinsic pathway is activated upon either spontaneous (due to over-expression of DR4 or DR5, or MADD knockdown) or ligand (i.e. TRAIL) induced oligomerization of DRs. This leads to the recruitment of FADD and procaspase-8 to the cytoplasmic domain of the DRs, and activation of caspase-8 followed by the activation of caspase-3 and cell death [1]–[3].

While MCF-7 and MDA-MB-231 showed significant levels of spontaneous apoptosis upon MADD knockdown, apoptosis noted in T47D cells, although higher, did not reach statistical significance (Fig. 3C). The T47D cells are inherently resistant to TRAIL, possibly due to expression of cFLIP_L_, which is a TRAIL resistance factor [22]. The cFLIP_L_ binds to FADD and prevents the recruitment of procaspase-8 and thus confers resistance to TRAIL-induced apoptosis [23]. Therefore, we did not include this cell line in mechanistic studies.

Because TRAIL induces apoptosis in cancer cells but not in their normal counterparts, it is an attractive candidate for cancer therapy. TRAIL and several agonistic antibodies that specifically bind to TRAIL receptors are currently being tested in clinical trials. A recent study demonstrated that the majority of breast cancer cell lines are very sensitive to TRAIL-induced apoptosis, suggesting that it could be used to treat recalcitrant breast cancers [24], [25]. However, a major concern for TRAIL-based therapies is the rapid induction of resistance which can lead to a more aggressive form of cancer [12], [26], [27]. The pro-survival role of MADD is limited to cancer cells and abrogation of MADD expression has no apparent effect on normal cell survival [28]. Therefore, knocking down MADD is likely to potentiate TRAIL-induced apoptosis selectively in cancer cells and significantly reduce the possibility of resistance development. Hence, we examined whether MADD knockdown could enhance TRAIL induced apoptosis in breast cancer cells.

MCF-7 cells were less sensitive to TRAIL-induced apoptosis at every concentration tested, whereas the more aggressive MDA-MB-231 cells were relatively more sensitive, which positively correlated with the dose of TRAIL used (data not shown). In order to test a possible synergistic effect between MADD knockdown and TRAIL treatment, we deliberately treated cells with a suboptimal dose of TRAIL and were able to induce much higher levels of apoptosis in both MDA-MB-231 and MCF-7 cells with MADD knockdown (Fig. 4).

All known pro-survival functions of MADD have been associated with the extrinsic apoptosis pathway involving the death receptors. However, the impact MADD knockdown might have on doxorubicin induced apoptosis, which primarily activates the intrinsic apoptosis pathway, has not been investigated. We had shown previously that over-expression of the pro-apoptotic isoform, IG20pa which can act as a dominant negative MADD rendered HeLa and PA-1 cancer cells more susceptible to chemotherapeutic drugs and γ-radiation- induced apoptosis [21], [29], [30]. Therefore, we investigated the combined effects of MADD knock down in breast cancer cells with doxorubicin treatment. Our studies showed that MCF-7 and MDA-MB-231 cells were relatively sensitive to low levels of doxorubicin (data not shown) and MADD knockdown enhanced doxorubicin-induced apoptosis in MCF-7 cells (Fig. 4A). Recent reports have shown that in breast and other cancer models, treatment with low doses of doxorubicin resulted in increased expression of TRAIL receptors, (i.e. DR4 and DR5) [6], [8], [31]. Therefore, we examined levels of DRs in doxorubicin-treated MCF-7 and MDA-MB-231 cells.

We found that upon treatment with a low dose of doxorubicin, the cell surface levels of DR4 and DR5 were increased in MCF-7 cells, while DR5 was increased in MDA-MB-231 cells (Fig. S2). This suggested that an increase in DR expression coupled with the absence of MADD expression led to enhanced extrinsic apoptosis (Fig. 5A) resulting from activation of DR associated caspase-8 (Fig. 5B). This is particularly interesting because mere over-expression of DRs can either lead to increased sensitization to TRAIL or enhanced spontaneous apoptosis due to receptor oligomerization even in the absence of ligand (TRAIL) binding [32].

MADD knockdown reduces the threshold for apoptosis as indicated by enhanced spontaneous death [5], [11], [13]. This lowered threshold combined with TRAIL or doxorubicin treatment may be enough to increase cell death. Since MADD negatively regulates activation of caspase-8, knockdown of MADD can result in enhanced activation of caspase-8 [4]. Moreover, addition of TRAIL or increased expression of DRs, due to doxorubicin treatment, likely magnified this effect through receptor oligomerization, which resulted in higher than expected levels of cell death. This notion was further supported by the reversal of enhanced apoptosis and increased DR associated caspase-8 activation upon DN-FADD expression (Fig. 5). Interestingly, it has been shown that in MCF-7 cells both DR-4 and DR5 engage the apoptotic pathway independent of the adaptor molecule FADD [33], [34]. Therefore, how DN-FADD could attenuate the activation of caspase-8 and apoptosis in these cells is not apparent and needs to be further investigated.

In summary, our results show that in addition to the enhancement of TRAIL-induced apoptosis, MADD knockdown could synergize the apoptotic effects of doxorubicin in breast cancer cells. These findings may have implications for developing novel strategies to treat breast cancer, overcome TRAIL resistance, and enhance conventional chemotherapies.

## Materials and Methods

### Antibodies and other reagents

Phycoerythrin (PE) conjugated anti-DR4 (DJR1 clone), anti-DR5 (DJR2-4 clone) anti-DcR1 (Phycoerythrin (PE) conjugated DJR3 clone), anti-DcR2 (DJR4-1 clone), anti-TRAIL (clone RIK 2), and an IgG isotype controlused for FACS analyses were obtained from eBioscience. Anti-DR5and caspase-8 (1C12) antibodies used in western blots were obtained from Cell Signaling. Human TRAILwas obtained from Peprotech Inc. Doxorubicinwas obtained from Sigma-Aldrich. An antibody (13L antibody) that specifically binds to a peptide encoded by exon 13L of *IG20* (SVRRRIYDNPYFEPQYGFPPEEDEDEQGESYTPRFSQHVSGNR), was generated as described before[14]. Dominant negative FADD (DN-FADD), a DED deletion plasmid construct as previously described [4], [35], was a kind gift from Dr. Vishva Dixit (Genentech, South San Francisco, CA).

### Preparation of tissue microarray slides

Tissue microarrays (TMA) containing 44 benign breast lesions, 39 ductal carcinoma *in situ* (DCIS) specimens and 86 malignant breast tumors were provided by Dr. Wiley. All the human tissue *sections were processed is from existing de-identified tissue samples only, and thus, this research qualified as a minimal risk to patients and their privacy, and approval for the use of these specimens with a exemption of consent was granted by the Institutional Review Board of the Office for the Protection of Research Subjects at University of Illinois at Chicago (Approval #: 2009-0081).*

### Immunohistochemical staining of breast cancer tissues

TMAs were incubated at 60°C for 1 hour, de-waxed, and immersed in alcohol. Sections were covered with a citrate-based antigen unmasking solution kits (Vector Laboratories) and subjected to microwave treatment for 10 minutes. Subsequently, the sections were incubated in 0.3% hydrogen peroxide for 30 minutes to inactivate endogenous peroxidase activity. Prior to application of the primary antibody, nonspecific interactions were blocked for 10 min using a blocking serum. Sections were incubated at 4°C overnight with an anti-MADD antibody at a 1∶800 dilution. The subsequent steps used the Vectastain Universal Quick kit (Vector Laboratories) and followed the manufacturer's instructions. Peroxidase staining was revealed with 3, 3-diaminobenzidine and then the sections were counterstained with Vector Hematoxilin QS. Sections were dehydrated, cleared and mounted in Vecta Mount mounting medium. Negative control staining was performed for each TMA slide without the primary antibody. Slides were scored for the intensity of staining in a semi-quantitative fashion independently by two pathologists: negative (0), weak (1+), intermediate (2+), or strong (3+).

### Cell culture

293T cells and MDA-MB-231 cells were cultured in Dulbecco's modified Eagle's medium (Invitrogen Life Technologies,) supplemented with 10% fetal bovine serum(Invitrogen), 2 mM L-glutamine(Invitrogen), 100 units/ml penicillin-G, and 100 µg/ml streptomycin(Invitrogen). MCF-7 cells were cultured in Minimal Essential medium (Invitrogen) supplemented with 5% fetal bovine serum, 100units/ml penicillin-G, 100 µg/ml streptomycin and 5.86% sodium bicarbonate(Invitrogen). Both MCF-7 and MDA-MB-231 cells were obtained from Dr. Jonna Frasor (Dept. of Physiology and Biophysics, UIC). T-47D cell line was obtained from Dr. Andrei Gartel (Department of Medicine, UIC). All these cell lines were original purchased from ATCC (Manassas, VA). These cells were maintained in RPMI (Invitrogen) supplemented with 10% fetal bovine serum, 100 units/ml of penicillin-G and 100 µg/ml of streptomycin. All cell lines were maintained at 37°C in a humidified atmosphere with 5% CO_2_.

### Immunofluorescence staining for MADD expression

MCF-7, MDA-MB-231 and T47D cells (3×10^5^) were placed into 60 cm culture dishes with cover glasses, and cultured overnight. The cells were fixed with acetone and permeabilized with 0.01% Triton X-100 followed by blocking with 1% BSA for 30 min at RT, Cells grown on cover slips were treated overnight with the rabbit anti-IG20 13L antibody at 4°C. Normal rabbit serum(Invitrogen) was used as a control. Subsequently, cells were stained with a biotinylated anti-rabbit antibody (Caltag Laboratories) and FTIC labeled streptavidin (Santa Cruz). The image was visualized and captured using LSM 510 META confocal microscope.

### Lentivirus production and transduction

As previously described [5], sub-confluent 293T cells in combination with a lentiviral vector, pcRev, pcTat, and pHIT/G were used to produce a self-inactivating replication-deficient lentivirus containing SCR-shRNA, 13L-shRNA, and 16E-shRNA sequences [5], [20], Each virus targets specific exons: 13L-shRNA targets exon 13L expressed only in IG20pa and MADD isoforms of the *IG20* gene, and 16E-shRNA targets exon 16 expressed only in IG20pa and IG20-SV2 isoforms of the *IG20* gene. The least amount of viral supernatant required to transduce 70% of target cells without apparent cytotoxicity was the optimal viral titer and was used in subsequent experiments.

Cells were plated at low density and when they cultures were 80% confluent, viral supernatant was added to the tissue culture dish and cultured for 4 hours. Warm culture medium was added to the culture dish and incubated for 24. GFP expression was monitored to confirm transduction efficiency.

### Reverse Transcription–Polymerase Chain Reaction

Total RNA was extracted from control and virus transduced cells using Trizol (Invitrogen), and 1 µg of RNA was used for reverse transcription–polymerase chain reaction (RT–PCR) using the Super-Script One-Step RT–PCR system (Invitrogen). cDNAs were synthesized at 50°C for 30 minutes followed by incubation at 94°C for 2 minutes. Subsequently, 35 cycles of PCR were carried out with denaturation at 95°C for 30 seconds, annealing at 55°C for 30 seconds and extension at 72°C for 1 minute; followed by a final incubation at 72°C for 7 minutes. The sequences of F2-B2 and GAPDH primers have been previously reported [36]. The PCR products were separated on a 2% agarose gel which allows for the identification of each of the four *IG20* isoforms.

### TMRM (tetramethylrhodamine methyl ester) staining

Cells positive for TMRM have intact mitochondria, and a reduction in TMRM staining served as a reliable marker for apoptosis. The MCF-7 (3.5×10^4^) and MDA-MB-231 (2×10^4^) cells were plated in 6-well plates and transduced with lentiviruses. Seventy-two hours post-transduction, cells were stained with 100 nM tetramethylrhodamine methyl ester (TMRM) (Molecular Probes-Invitrogen) for 15 minutes at 37°C. Cells were collected, washed with cold PBS and analyzed using a BD FACS Calibur (BD Biosciences). Only GFP-positive (shRNA-expressing) cells indicating lentivirus expression were included in the analysis.

### Crystal violet staining

MCF-7 (2×10^4^) and MDA-MB-231 (1×10^4^) cells were plated into 6-well plates. Twenty-four hours later, cells were treated with different shRNA-expressing lentiviruses for 4 hours followed by the addition of fresh warm medium. The medium was replaced every 3 days. When control wells were confluent, they were fixed in ice-cold methanol and stained with crystal violet to assess viability and colony formation.

### Trypan blue exclusion

MCF-7 (2×10^4^) and MDA-MB-231 (1×10^4^) cells were plated into 6-well plates. Twenty-four hours later, cells were treated with different shRNA-expressing lentiviruses for 4 hours and then replenished with fresh warm medium. Cells were counted 24 hours post-transduction and counted again on days 3 and 5. Cell death was determined by Trypan Blue (Invitrogen) exclusion.

### FACS analysis to detect cell surface expression of receptors

At various times after treatment, cells were collected in enzyme-free cell dissociation buffer (Invitrogen), washed once with PBS containing 0.5% bovine serum albumin, and allowed to stand in the same buffer for 10 minutes at 4°C. PE-conjugated antibodies were used to stain samples for 30 minutes at 4°C. A mouse IgG antibody was used as an isotype control. Cells were washed with PBS and analyzed using a BD FACS Calibur (BD Biosciences).

### Immunoblot analysis

Cells were lysed for 30 min at 4°C in a lysis buffer (20 mM Tris pH 7.5, 2 mM EDTA, 3 mM EGTA, 2 mM dithiothreitol [DTT], 250 mM sucrose, 0.1 mM phenylmethylsulfonyl fluoride, 1% Triton X-100) containing a protease inhibitor cocktail (Sigma-Aldrich) and phosphatase inhibitor (Sigma-Aldrich). Protein concentrations were determined using Bradford assay (Bio-Rad). Protein samples (50 ug per lane) were separated by SDS-PAGE using a 10% gel and transferred to nitrocellulose membranes, blocked with 5% skim milk and probed using corresponding primary antibodies, followed by horseradish peroxidase-conjugated secondary antibodies. The protein bands were visualized by enhanced chemiluminescence.

### Statistical analysis

One-way Anova, the Tukey-Kramer test (for Fig-1) or the student's t-test (for the remaining figures) was used to compare groups as indicated. A P-value of 0.05 was considered significant. Analysis was performed utilizing GraphPad Prism 5 statistical software and Microsoft Excel Software (version 2003). Results are expressed as mean ± SE.

## Supporting Information

Figure S1 **MADD knockdown leads to decreased breast cancer cell survival.** Upon MADD knockdown MCF-7 **(A)** and MDA-MB-231 **(B)** cells were plated in 6-well plates, transduced with indicated viruses and live cells were counted at indicated days after transduction using trypan blue. Data shown are representative of three different experiments. MCF-7 **(C)** and MDA-MB-231 **(D)** cells were cultured until control wells were fully confluent. At that time the cells were fixed with ice cold methanol and stained with crystal violet.
(TIF)

Figure S2 **Doxorubicin treatment over time results in enhanced cell surface expression of death receptors.** At each time point (0, 24, 48, 72 hours), MCF-7 **(A, C)** and MDA-MB-231 **(B, D)** cells were collected and stained with PE conjugated antibodies to DR4 **(A, B)** and DR5 **(C, D)** or an IgG isotype negative control antibody and analyzed by FACS, P<0.05 *vs.* un-treated group. Data are shown as the fold change in the absolute fluorescence intensity at various time points compared to zero time point. Summarized data from three independent experiments are shown.
(TIF)
