# Supplementary figures and images for: MADD Knock-Down Enhances Doxorubicin and TRAIL Induced Apoptosis in Breast Cancer Cells
Source: PLoS One. 2013 Feb 15;8(2):e56817. doi: 10.1371/journal.pone.0056817 (PMC3574069; doi:10.1371/journal.pone.0056817)

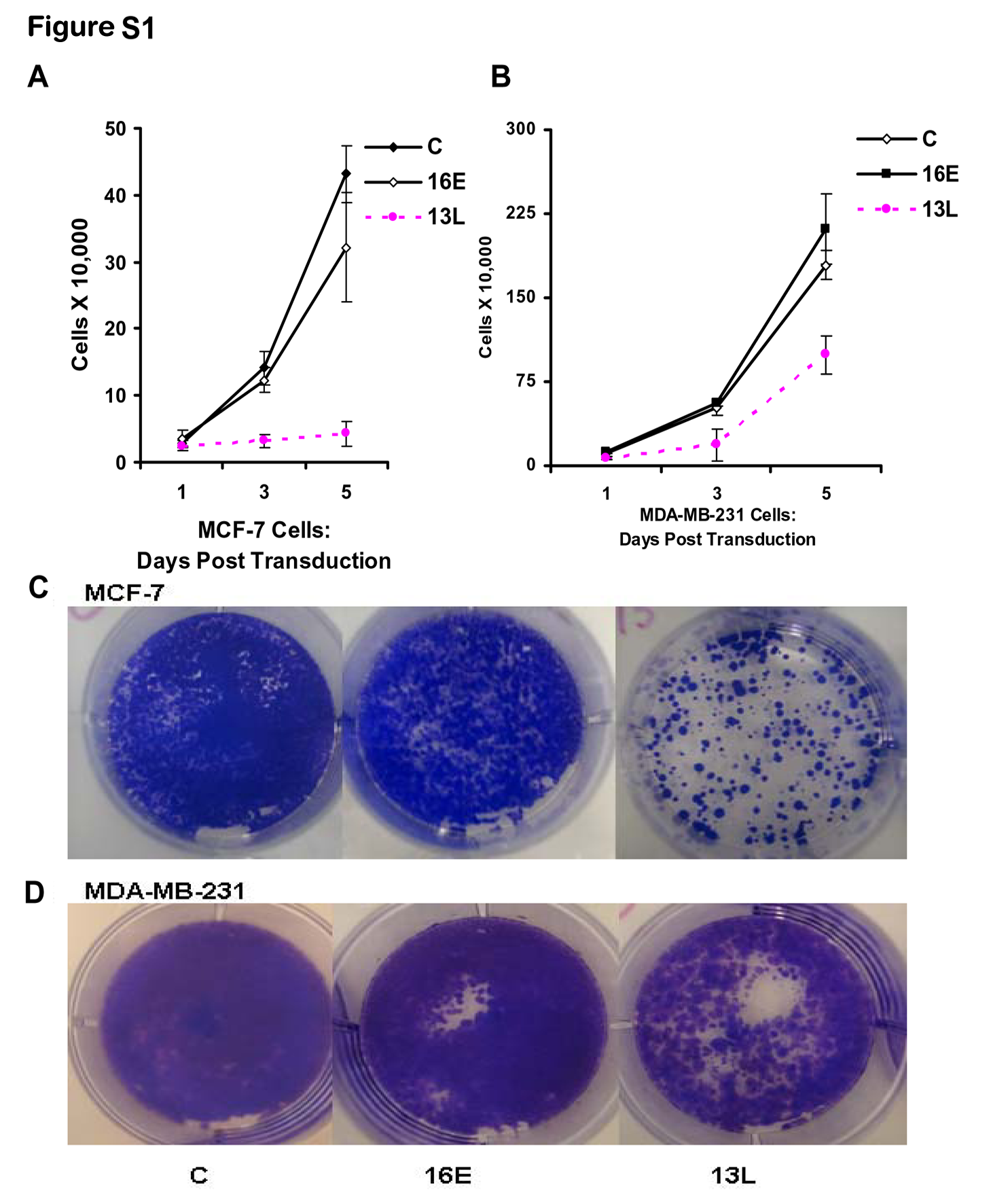

Supplement: Figure S1 — MADD knockdown leads to decreased breast cancer cell survival. Upon MADD knockdown MCF-7 (A) and MDA-MB-231 (B) cells were plated in 6-well plates, transduced with indicated viruses and live cells were counted at indicated days after transduction using trypan blue. Data shown are representative of three different experiments. MCF-7 (C) and MDA-MB-231 (D) cells were cultured until control wells were fully confluent. At that time the cells were fixed with ice cold methanol and stained with crystal violet. (TIF) [file pone.0056817.s001.tif]

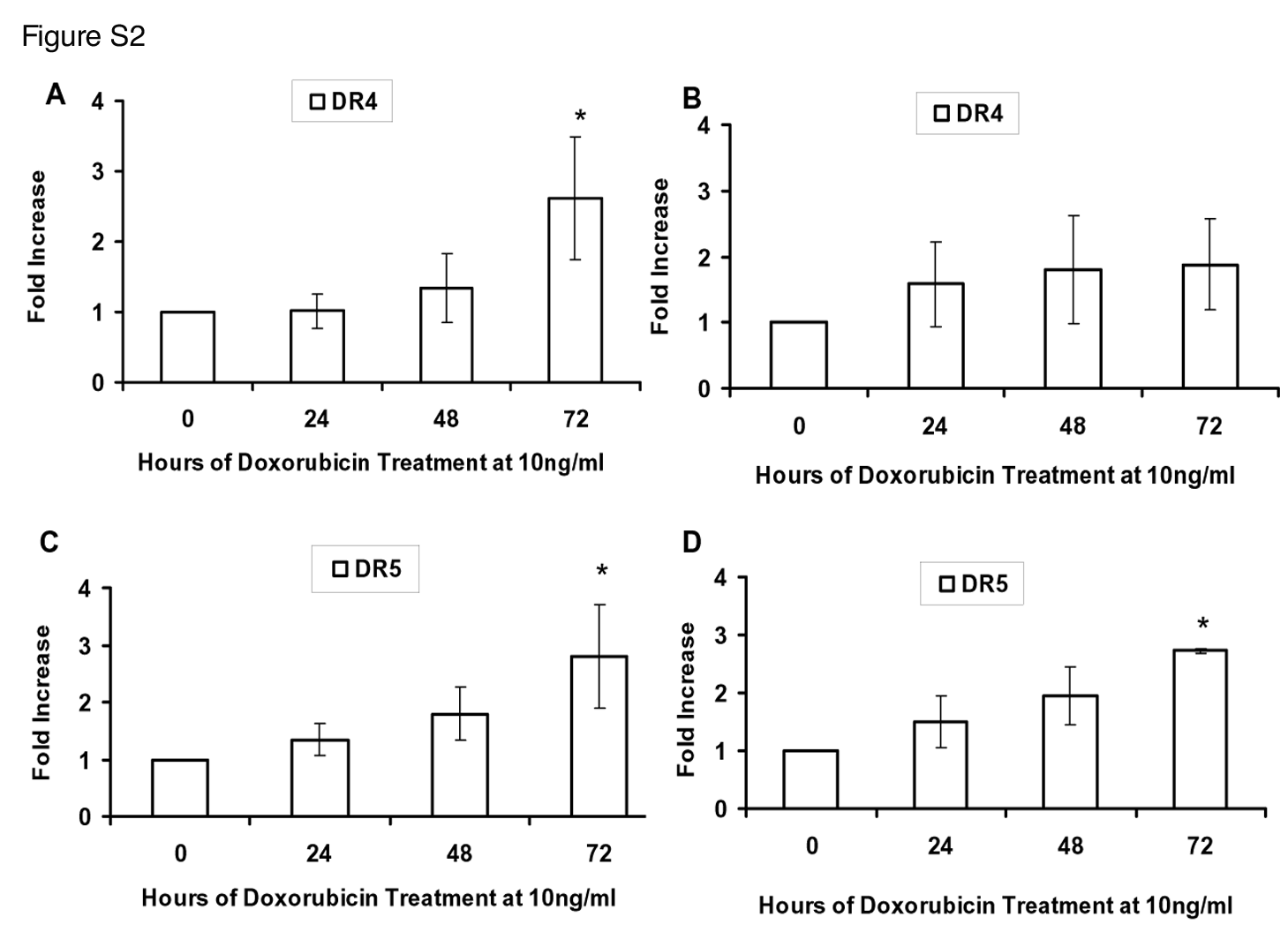

Supplement: Figure S2 — Doxorubicin treatment over time results in enhanced cell surface expression of death receptors. At each time point (0, 24, 48, 72 hours), MCF-7 (A, C) and MDA-MB-231 (B, D) cells were collected and stained with PE conjugated antibodies to DR4 (A, B) and DR5 (C, D) or an IgG isotype negative control antibody and analyzed by FACS, P<0.05 vs. un-treated group. Data are shown as the fold change in the absolute fluorescence intensity at various time points compared to zero time point. Summarized data from three independent experiments are shown. (TIF) [file pone.0056817.s002.tif]
